# PROFILE OF OLDER PUBLIC TRANSPORTATION USERS IN THE UNITED STATES: IMPLICATIONS FOR AGE-FRIENDLY COMMUNITIES

**DOI:** 10.1093/geroni/igac059.1245

**Published:** 2022-12-20

**Authors:** Afnan Gimie, Andrea Melgar Castillo, C Daniel Mullins, Jason Falvey

**Affiliations:** University of Maryland School of Medicine, Baltimore, Maryland, United States; University of Maryland School of Pharmacy, Baltimore, Maryland, United States; University of Maryland School of Pharmacy, Baltimore, Maryland, United States; University of Maryland Baltimore, Baltimore, Maryland, United States

## Abstract

Public buses, trains, and trams are a growing mode of transportation for older adults in the United States, yet many environmental and health related barriers to use have been reported. Characterizing the population of older public transit users is essential for developing age-friendly communities. We used data from 5696 urban, community dwelling older adults in round 5 of the National Aging and Trends Study (NHATS), an annual nationally representative survey of late-life disability. Using SAS (version 9.4), weighted frequencies were calculated and compared between public transit and non-transit users using procedures that account for the complex design of the NHATS survey. Compared to non-transit users, those who reported using transit within the last month (n=555, 9.8%; weighted n=3,122,583) were significantly more likely to identify their race/ethnicity as Black or Hispanic (50% vs 28%) and reported difficulty meeting financial needs for housing, utility, and food (12% vs 7%), and to speak a language other than English (14% vs 8%). Transit users were significantly less likely to use a walker (9% vs 14%) or wheelchair/scooter (4% vs 9%). Additionally, 15% of transit users did not have a working cell phone and 42% did not have a working computer. Over 20% of transit users (weighted n=658,850) rely on these services to get to their doctor. These findings highlight the clinical, social, and financial barriers that disproportionately affect over 3 million older adult transit users in the United States, and inform initiatives oriented towards improving community access for older adults.

